# A population-based evolutionary search approach to the multiple minima problem in de novo protein structure prediction

**DOI:** 10.1186/1472-6807-13-S1-S4

**Published:** 2013-11-08

**Authors:** Sameh Saleh, Brian Olson, Amarda Shehu

**Affiliations:** 1 Department of Computer Science, George Mason University, 4400 University Dr., Fairfax, VA, 22030, USA; 2 Department of Bioengineering, George Mason University, 4400 University Dr., Fairfax, VA, 22030, USA; 3 School of Systems Biology, George Mason University, 10900 University Blvd., Manassas, VA, 20110, USA

## Abstract

**Background:**

Elucidating the native structure of a protein molecule from its sequence of amino acids, a problem known as de novo structure prediction, is a long standing challenge in computational structural biology. Difficulties in silico arise due to the high dimensionality of the protein conformational space and the ruggedness of the associated energy surface. The issue of multiple minima is a particularly troublesome hallmark of energy surfaces probed with current energy functions. In contrast to the true energy surface, these surfaces are weakly-funneled and rich in comparably deep minima populated by non-native structures. For this reason, many algorithms seek to be inclusive and obtain a broad view of the low-energy regions through an ensemble of low-energy (decoy) conformations. Conformational diversity in this ensemble is key to increasing the likelihood that the native structure has been captured.

**Methods:**

We propose an evolutionary search approach to address the multiple-minima problem in decoy sampling for de novo structure prediction. Two population-based evolutionary search algorithms are presented that follow the basic approach of treating conformations as individuals in an evolving population. Coarse graining and molecular fragment replacement are used to efficiently obtain protein-like child conformations from parents. Potential energy is used both to bias parent selection and determine which subset of parents and children will be retained in the evolving population. The effect on the decoy ensemble of sampling minima directly is measured by additionally mapping a conformation to its nearest local minimum before considering it for retainment. The resulting memetic algorithm thus evolves not just a population of conformations but a population of local minima.

**Results and conclusions:**

Results show that both algorithms are effective in terms of sampling conformations in proximity of the known native structure. The additional minimization is shown to be key to enhancing sampling capability and obtaining a diverse ensemble of decoy conformations, circumventing premature convergence to sub-optimal regions in the conformational space, and approaching the native structure with proximity that is comparable to state-of-the-art decoy sampling methods. The results are shown to be robust and valid when using two representative state-of-the-art coarse-grained energy functions.

## Background

Obtaining a detailed structural characterization of the biologically-active (native) state of a protein molecule holds great promise for unraveling the relationship between protein structure and function and is key to our understanding and treatment of disease. Elucidating a representative three-dimensional structure of the protein native state, referred to as the native structure, is essential in structure-driven studies on engineering novel proteins, predicting protein stability, modeling interactions upon protein complexation, and designing effective drugs [1-9]. While experimental techniques, such as X-ray crystallography, nuclear magnetic resonance, and cryo-electron microscopy, are able to extract atomic coordinates of the native structure, these techniques are laborious, expensive, and have limitations either in the subset of proteins on which they can be applied or in the resolution and quality of their findings. As a result, they have not kept up with the exponential growth in protein sequences in the genome sequencing era [10]. For instance, currently, only in about half of all well-characterized protein families do we have native structures for all family members [11]. In light of this, computational approaches are necessary to model the protein native state.

When a protein of interest has sequence homologs among other proteins of known native structure, a template for its native structure can be easily obtained with homology-based methods. When this is not the case, the task falls on de novo approaches to compute the native structure mainly from knowledge of the protein's amino-acid sequence [12]. The foundation of de novo approaches is due to Anfinsen's experimental demonstrations that the amino-acid sequence of a protein determines to a great extent the three-dimensional native structure where the sequence is biologically active [13]. However, finding this structure among the various spatial arrangements, or conformations, that can be assumed by the chain of amino acids in a protein, remains a central challenge in computational structural biology [14,15].

**Figure 1:**
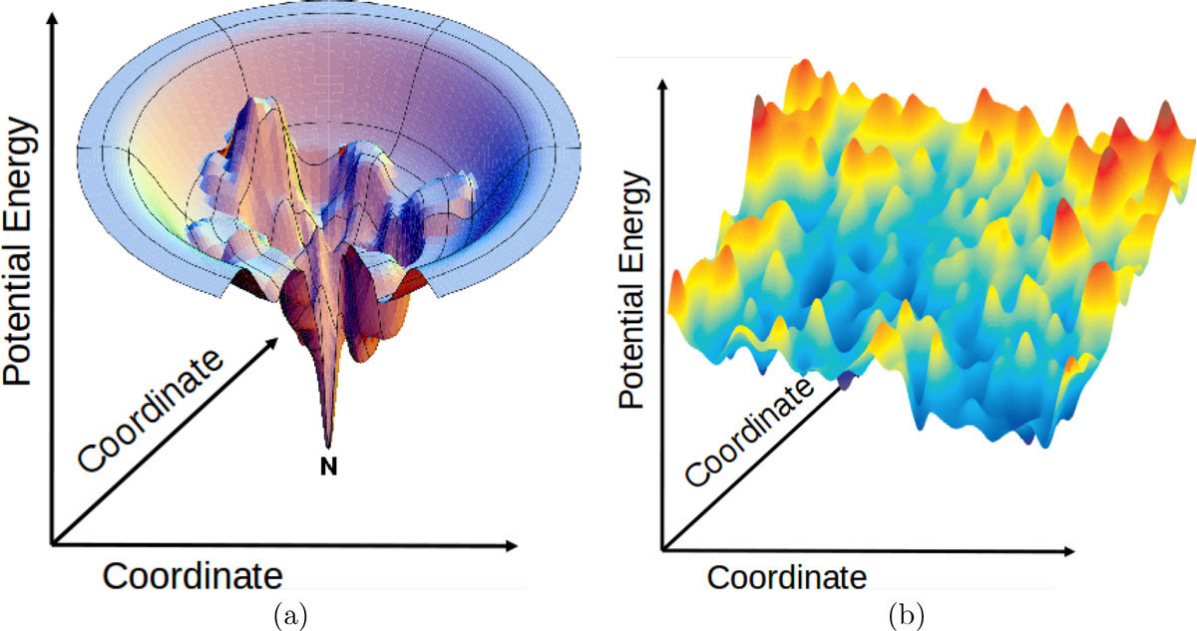
**The native state, labeled N, is associated with the free-energy minimum and is expected to consist of conformations located at the deepest unique basin of the protein energy surface **[16]. In contrast to this classic illustration, adapted from [16], the illustration to the right shows that the actual surface reconstructed by current energy functions is often weakly-funneled, rich in local minima, and with non-native states in deeper basins than the native state.

The majority of de novo approaches do not model the physical process of folding in which the protein chain gradually transitions from its unfolded to the folded or native state [12]. Instead of a kinetics view, most approaches exploit the thermodynamics view, which posits that the native state is that of lowest free energy and is in itself an ensemble of conformations [13]. While the totality of atomic interactions in a conformation results in a potential energy value and allows associating an energy surface [16,17] with the protein conformational space (illustrated in Figure 1(a)), the notion of free energy allows organizing the conformational space and its energy surface into states. The vertical axis of the energy surface, as illustrated in Figure 1(a), records the potential energy (or the "internal free energy") of a conformation, whereas the lateral axes represent the many underlying dimensions. The lateral width of this surface denotes entropy, or the degree of conformational diversity while the chain maintains a given potential energy value. The notion of free energy is captured in F = E - TS, where F is free energy, E is potential energy, T is temperature, and S is entropy of a state or ensemble of conformations. Under a statistical mechanics treatment, the native state of a protein sequence is a conformational ensemble of lowest free energy [16,17]. The native structure is a representative of this ensemble and is only effective when the ensemble is structurally homogeneous.

In the early days of de novo protein structure prediction, the operating assumption was to consider the protein native state to be a homogeneous conformational ensemble of negligible structural diversity. This justified disregard of entropy. Instead, the objective of many optimization techniques devised for de novo structure prediction became finding the global minimum of the potential energy surface (coined by Scheraga and colleagues as the Global Minimum Energy Conformation, or GMEC) [18]. Finding the GMEC is an NP-hard problem, mainly because the size of the space of all possible conformations of a protein chain grows exponentially with the length of the chain [19]. This makes deterministic or combinatorial search infeasible and demands instead powerful probabilistic search and optimization algorithms [20].

To add to the complexity, while the true protein energy surface is funnel-like, as illustrated in Figure 1(a), the actual surface probed with current potential energy functions, even state of the art ones, can be quite different. As Figure 1(b) illustrates, the energy surface available to a search algorithm is often weakly funneled, with many non-native states competing or even characterized by lower conformational energies than the native state [21]. This surface is rich in local minima, many of which are artifacts introduced by inherent inaccuracies in computationally-feasible potential energy functions for protein systems [22].

The high dimensionality of the protein conformational space notwithstanding, the issue of multiple minima is particularly troublesome. To address this issue, frameworks for de novo structure prediction do not seek the GMEC. Instead, they aim to obtain a broad view of the low-energy regions in the probed energy surface through an ensemble of low-energy (decoy) conformations. Since this ensemble is expected to contain many non-native conformations with lower energies than native or near-native conformations (the term near-native aims to include conformations within the native basin), retaining conformational diversity in this ensemble is key to increasing the likelihood that the native state has been captured.

The predominant framework in de novo modeling is to split the task into two stages. Stage one obtains the decoy ensemble typically by launching multiple Metropolis Monte Carlo (MMC) [23] trajectories, each mapping some initial conformation (possibly sampled at random) to a local minimum. In order to obtain a diverse ensemble in a feasible amount of time, side chains are typically sacrificed at this stage, and a coarse-grained representation mainly of the backbone is employed instead for the protein chain. Once the decoy ensemble is obtained, the decoys are grouped by structural similarity through clustering-based techniques in order to remove redundancy and reduce the size of this ensemble for the next stage. Long heavy-duty energetic refinement in all-atom structural detailed is conducted in the second stage from each retained decoy. The hope is that if one of these is in the vicinity of the true native basin, heavy-duty refinement in the second stage will yield convergence to the native state [6,24-29].

In this paper we present a novel stochastic optimization approach for decoy sampling in de novo protein structure prediction. The proposed approach is based on evolutionary computing (EC) to effectively address the high-dimensional protein conformational space and the rugged potential energy surface. Given the challenges, decoy sampling methods often draw inspiration from different fields. Typical stochastic optimization strategies to better navigate the conformational space include many enhancements of the basic MMC approach, such as simulated annealing, parallel tempering, local energy flattening, and more (cf. to Ref. [15]). Yet others build on evolutionary search approaches, such as Basin Hopping (BH) [30] and more [31]. Indeed, many decoy sampling methods find their origins in the EC community. On the other hand, the most successful de novo structure prediction protocols nowadays incorporate domain-specific knowledge originating in the computational structural biology community. The most salient developments in this community include the design of both coarse-grained energy functions and protein chain representations and the use of the molecular fragment replacement technique to simplify the conformational space and energy surface.

It is natural to propose an evolutionary search approach for decoy sampling. Evolutionary Algorithms (EA) are powerful stochastic optimization algorithms proposed by the EC community to address difficult search and optimization problems. Some preliminary work has been conducted in this community on de novo protein structure prediction [32-38]. In many of these studies, an EA explores the protein conformational space by evolving a representative set or population of conformations towards lowest potential energy. A Memetic EA (MEA) combines the global EA search with a short minimization phase. In MEA, each move at the global level is accompanied by a projection to a nearby local minimum, effectively allowing the algorithm to explicitly probe local minima in a rugged energy surface. However, the majority of EAs devised for decoy sampling fail to make use of state-of-the-art domain-specific techniques, such as coarse graining and molecular fragment replacement. These techniques have been proven effective in the computational structural biology community, though in the context of MMC-based methods. In addition, in current EAs for de novo structure prediction, the predominant goal is to find the GMEC rather than examine how well the native structure is actually recovered. As a result, studies employing evolutionary strategies and EAs for de novo structure prediction are typically restricted to applications on very small molecules or toy models.

In this paper we propose an evolutionary search approach to address the multiple-minima problem in decoy sampling for de novo structure prediction. Two population-based EAs are presented that follow the basic approach of treating conformations as individuals in an evolving population. The contribution of the work presented here is two-fold. The first contribution is the combination of evolutionary search strategies with state-of-the-art coarse-grained representations, sophisticated energy functions, and the molecular fragment replacement technique. Our results make the case that such combination allows a rather basic EA to generate useful decoys for the first stage of de novo structure prediction and not be limited to toy models. Indeed, the domain of applications is extended to small- to medium-size proteins, and results are shown to be comparable to other popular decoy sampling methods in computational structural biology.

We conduct a detailed analysis and show in realistic case studies that the simple EA is highly exploitative. While it may sample conformations in proximity to the native structure, the greedy nature of the algorithm makes it prone to converge prematurely to sub-optimal minima. This conclusion further makes the case that, while traditional optimization algorithms in the EC community focus on finding the GMEC, this goal is not sufficient in the context of de novo structure prediction. Moreover, it is now well understood that even state of the art energy functions for protein modeling allow reconstructing energy surfaces where near-native conformations are often not associated with the lowest-energy minima [21,22]. The issue has been well-studied in the computational structural biology community [39,40] and is the main reason why de novo structure prediction methods aim to obtain a broad view of the energy surface by generating many structurally-diverse low-energy decoys.

Therefore, a second contribution of this paper is to propose an MEA to obtain a diverse ensemble of low-energy decoys that represent low-energy minima in the protein energy surface. Here we show that the MEA is capable of retaining structural diversity in its decoy ensemble. Our analysis shows that the MEA is less prone to convergence than the EA. The results are shown to be robust and valid when guiding the algorithms with two representative coarse-grained energy functions commonly used in state-of-the-art de novo structure prediction protocols.

We note that a preliminary implementation of the algorithms proposed here have been presented in [41]. In this work we present a more general setup for the algorithms and conduct a more detailed analysis, part of which additionally compares guidance by two state-of-the-art energy functions.

The rest of this paper is organized as follows. In the Methods section we describe the proposed EA and MEA. Results presented in the Results section show how the addition of a minimization step in MEA improves the ability to sample conformations near the native state. Analysis also quantifies the diversity of the decoy ensemble obtained through the MEA over that obtained through the EA. The employment of two different coarse-grained energy functions allows drawing interesting observations on current energy functions and their ability to guide an algorithm of enhanced sampling capability to the native basin. Results are finally compared to those obtained by representative MMC-based methods developed by us and others for decoy sampling. Findings and future research directions are summarized in the Conclusions section.

## Methods

Since the evolutionary search approach proposed here makes use of two important developments in the de novo structure prediction community - the use of coarse graining (and coarse-grained energy functions to operate on coarse-grained conformations) and the molecular fragment replacement technique - these are summarized first. We then discuss the main ingredients of our approach followed by details of the EA and MEA proposed in this paper. We note that a preliminary version of this work has appeared in [41]. While we reserve greatest detail for novel algorithmic components presented in this work, we summarize the entire method for the sake of completeness.

### Coarse-grained representation

As is commonly done in de novo structure prediction, the representation employed here mainly models the backbone of a protein chain and largely sacrifices structural details of the side chains in interest of efficiency. This approach relies on the availability of accurate and fast side-chain packing techniques [42,43] that are then able to pack side chains on promising coarse-grained decoys before the longer refinements in the second stage of de novo protocols. Since two different state-of-the-art coarse-grained energy functions are considered and compared here for their ability to guide the search to near-native conformations, two slightly different coarse-grained representations are used in each setting. In both cases, the *N, C, C_α_*, and *O *heavy backbone atoms are modeled explicitly. When using AMW, side-chains are represented only with their *C_β _*atom (except for glycine). When using Rosetta, a side chain is represented with a pseudo-atom at its centroid.

Both coarse-grained energy functions operate on cartesian coordinates of modeled atoms to associate a potential energy value or score with a conformation. The internal representation employed by the algorithms proposed here is angular and maintains only three backbone dihedral angles (*φ, ψ, ω*) per amino acid. This is also known as a kinematic model and is based on the idealized geometry assumption, which fixes bond lengths and angles to idealized (native) values (taken from CHARMM22 [44]) and limits variations to backbone dihedral angles. The summary of the molecular fragment replacement technique below describes how this representation is used to obtain conformations. After a conformation is obtained in its angular representation, forward kinematics is employed to obtain cartesian coordinates for the modeled atoms from the backbone dihedral angles [45]. This angular representation is commonly used in de novo structure prediction protocols, including Rosetta [46], and is an efficient representation that reduces the dimensionality of conformational space to 3*n *dimensions for a chain of *n *amino acids.

### Coarse-grained potential energy function

Our experiments in this paper consider two state-of-the-art coarse-grained energy functions, a modified version of the Associative Memory Hamiltonian with Water (AMW), and the Rosetta energy function, described below.

#### AMW energy function

AMW has been originally proposed in [47]. It has been used in its original form and in adaptations by us and others in the context of MMC-based decoy sampling methods [24,48-56]. Our adaptation of AMW sums 5 non-local terms (local interactions are not modeled, as bond lengths and bond angles are kept at ideal values under the idealized geometry assumption): *E*_AMW _= *E*_Lennard*−*Jones _+ *E*_H*−*Bond _+ *E*_compaction _+ *E*_burial _+ *E*_water_. The *E*_Lennard*−*Jones _energy term is implemented after the 12-6 Lennard-Jones potential in AMBER9 [57] allowing a soft penetration of van der Waals spheres. The *E*_H*−*Bond _term allows modeling hydrogen bonds and is implemented as in [58]. The other terms, *E*_compaction_, *E*_burial_, and *E*_water_, allow formation of a hydrophobic core and water-mediated interactions (we direct the reader to Ref. [24] for further details on this potential energy function).

#### Rosetta energy function

The second coarse-grained energy function we employ here corresponds to the *score3 *setting in the suite of energy functions used in the Rosetta de novo protocol and package [46]. The different energy functions used in the actual Rosetta de novo protocol are scaled versions of a full energy function. The full function is a linear combination of 10 terms that measure repulsion, amino-acid propensities, residue environment, residue pair interactions, interactions between secondary structure elements, density, and compactness. The different substages used in the Rosetta protocol use subsets of the terms of the full energy function and modify weights in the linear combination to gradually promote certain interactions over others in the course of the protocol. We use here the *score3 *setting, as this corresponds to the full coarse-grained Rosetta energy function.

### Molecular fragment replacement technique

The employment of the molecular fragment replacement technique originates in [59]. In the context of an evolutionary search approach, this technique is used as follows. A residue position *i *is sampled uniformly at random over the chain of the parent conformation. A fragment [*i, i *+ 2] of 3 consecutive amino acids is then defined over the chain. The 9 *φ, ψ, ω *backbone dihedral angles corresponding to this fragment in the parent conformation are then replaced with a configuration of 9 angles sampled uniformly at random over configurations recorded for that fragment in a fragment configuration library. The library stores fragment configurations extracted from known protein native structures. Therefore, the modifications are more likely to result in physically-realistic child conformations. More details on the construction of fragment configuration libraries can be found in [51].

### Overview of main ingredients in the proposed evolutionary search approach

The basic EA is shown in pseudo-code in Algorithm 1. In summary, an evolutionary computing model is employed for optimization of a given protein energy function (fitness function). Under this framework, conformations are ever-improving solutions of the fitness function. A given and fixed number PopSize of conformations are stored in a population *P*, which is initialized to consist of random conformations for a given protein sequence *α *at generation 0 (line 1 in Algorithm 1). *P *evolves over a series of iterations or generations to obtain better solutions to the fitness function (lines 2-6). We note that in the typical EA, the goal is to maximize fitness. Since here the fitness function is potential energy, the goal is to minimize fitness. In this work, we maintain a constant capacity PopSize through each generation for the evolving population *P*. As line 3 shows, the conformations in the current generation serve the role of parents to generate new child conformations *C*. Parents and children compete for survival (line 4). Comparison of fitness values is used for this purpose. Surviving conformations make up the evolved population in the next generation and are added to the running ensemble Ω*_α _*of (decoy) conformations analyzed in the Results section. We now relate details on each of the components of the basic EA and its modification to obtain the MEA that additionally incorporates minimization.

**Algo. 1 **Algorithm 1: Pseudo code is shown for a canonical EA

    **Input: **Amino acid sequence, population size, and number of offspring as *α*, PopSize, and NumChild.

    **Output: **the set of sampled of conformations, Ω*_α_*

1: P *← *INITIALPOPULATION(PopSize)

2: **while **Eval*_count _<*Eval*_max _***do**

3:    *C ← *SAMPLECONFORMATIONS(*P*, NumChild)

4:    *P_new _←*SELECTPOPULATION(*P, C*)

5:    Ω_α _← Ω_α _∪ *P_new_*

6:    *P ← P_new_*

### A basic EA to sample low-energy protein conformations

In each generation in the basic EA, parent conformations are first selected from the current population *P*. Fitness-proposal selection is used, whereby a linear weighting of fitness values (energy functions) is used to associate a higher probability of selection to conformations with lower energies. The selected parent conformations are then modified through the molecular fragment technique summarized above to obtain NumChild child conformations. The result of this process over the generations is that lower-energy conformations will be selected more often to spawn children (a parent can be selected more than once).

The basic EA summarized in pseudo-code in Algorithm 1 evolves a fixed-size population *P*. To select a new population *P_new_*, the current population *P *is combined with the set of newly-sampled children *C*. Truncation selection is then used to reduce the merged set back to *|P| *conformations. In truncation selection, conformations are ordered according to their fitness values (energy values here), and the *|P| *conformations with the lowest energies are selected to constitute the next population. This selection technique is highly exploitative.

The molecular fragment replacement technique summarized above is employed to modify selected parent conformations to obtain child conformations. The technique is also used to provide the EA with a diverse set of low-energy conformations in its initial population at generation 0. Starting from an extended conformation, *n − *2 fragment configuration replacements are conducted over the protein chain to obtain a random but realistic conformation. This process is repeated *|P| *times to effectively obtain *|P| *conformations that constitute the initial population and thus seed the basic EA.

### A memetic EA to sample local minima

As described, the basic EA conducts a highly exploitative search, progressing towards lower-energy conformations in pursuit of the GMEC. As discussed in the Background section, the goal in de novo structure prediction is instead to search for a diverse set of sufficiently low-energy decoy conformations rather than the absolute minimum due to approximations and resulting inaccuracies in calculations of potential energy. Due to this exploitative nature, the population of decoys (all child conformations in growing ensemble) in the basic EA risks premature convergence to narrow low-energy basins that may be far from the native state in conformational space. As a result, further exploration and discovery of useful decoy conformations can be severely limited in further generational propagation in the basic EA.

We investigate an MEA to enhance sampling of near-native decoy conformations. The MEA allows for explicit sampling of local minima in the protein energy surface. An additional minimization step is employed in the MEA to map a child conformation sampled by the basic EA to a nearby local minimum. We employ an efficient greedy local search to implement the minimization step. The greedy search performs a series of fragment replacements only accepting modifications that decrease potential energy. This continues until *n *consecutive fragment replacements are rejected (*n *is the length of the target protein), effectively indicating that a local minimum has been reached. The resulting conformation representing the minimum replaces the initial conformation and is added to the set *P*_new _of child conformations.

Child conformations that survive the truncation selection and therefore become members of the evolved population in the next generation are representatives of local minima in the energy surface. In the next iteration, some of them may be selected to be parents. When that happens, a fragment configuration replacement applied to one of them (that is part of the process to generate new children) is equivalent to a jump out of the current minimum represented by the parent conformation. This *resetting *is crucial to obtain new nearby minima in the energy surface and so reduce the exploitative nature of the basic EA. The resetting helps enhance conformational diversity in the MEA, as it essentially jumpstarts the exploration with new starting points for the ensuing minimization.

The definition of a local minimum employed here approximates a true local minimum in the energy surface. Previous work in the context of a basin hopping trajectory [54,60] shows that, at a coarse-grained level of detail, this working definition is sufficient to sample low-energy decoy conformations near the native state.

We further note that the number of generations in either the basic EA or MEA are not fixed a priori. Instead, each algorithm runs for a fixed budget of energy evaluations. In the memetic version, each energy evaluation within the minimization contributes to the overall count of energy evaluations in the fixed budget.

### Analysis of structural diversity in conformational ensembles

While the majority of the analysis detailed in the Results section employs energy values and proximity of computed decoys to the known native structure, the ensembles obtained through the EA and MEA are directly compared to each other employing a simple lRMSD-based measure. Essentially, pairwise lRMSDs between any two conformations in a given population are computed, and the median lRMSD measuring the structural diversity in the population is recorded. The median lRMSD is then plotted in the Results section over all populations in the EA versus the MEA algorithm to visualize how structural diversity is retained or lost by any of these algorithms.

## Results

The basic EA and MEA are each run on a testbed of 15 protein sequences with known native structures. The results presented below focus on comparing the sampling ability between the two algorithms with respect to obtained lowest energy and proximity to the native structure. Results obtained by MEA are compared to three other state-of-the-art MMC-based methods. Finally, the results obtained by the MEA using the AMW energy function are compared with those obtained using the Rosetta *score3 *energy function. The results show that the incorporation of domain-specific knowledge in EA makes this simple algorithm competitive. However, the addition of the minimization step in MEA significantly improves the ability to sample near-native conformations and makes MEA competitive to other state-of-the-art decoy sampling methods. Moreover, when using the Rosetta over the AMW energy function, the results improve further for a large subset of proteins.

### Experimental setup

We set population size *|P| *to be 1000 conformations. This is large enough to maintain a structurally-diverse population but small enough to ensure competition among members in a population. In MEA, the number of child conformations sampled at each generation, numChild, is set at 250. Note that this is less than population size, which allows limiting competition among children and in turn increasing the percentage of children that survive and are passed on to the next generation. In the basic EA, numChild = 4000. Since EA does not minimize each conformation, the majority of children tend to have high potential energies. Generating a larger number of children in the basic EA increases the chance of obtaining some low-energy children, and in turn allowing for downward exploration of the energy surface with each generation.

We execute each algorithm 3 times, each time using a fixed budget of 10 million energy evaluations. The purpose for fixing this budget rather than number of generations is as follows. The most computationally intensive task that takes up more than 90% of runtime of either EA or MEA is the calculation of potential energy for each conformation.

By fixing the number of such evaluations we maintain a fair comparison between the algorithms and across a broad range of target protein systems. In practice, depending on the length of the target protein, each experiment takes 30*−*50 hours of CPU time on a 2.4Ghz Core i7 processor.

The majority of the analysis below that compares which algorithm comes closer to the native structure or how often it does so relies on calculating the least Root Mean Square Deviation (lRMSD) to the known native structure for each conformation in the output ensemble Ω*_α _*(Ω*_α _*is the union of the populations in each generation). lRMSD stands for least RMSD, because it is preceded by an alignment step that finds a rigid-body transformation minimizing the RMSD between two conformations post alignment. Post-alignment RMSD is measured as a weighted Euclidean distance, summing distances between corresponding *C_α _*atoms. While not a Euclidean metric, lRMSD is a dissimilarity metric. A lower lRMSD value between a conformation and the sought native structure means that the conformation is closer to the native structure. A higher lRMSD, however, does not necessarily mean significant structural differences, as lRMSD is not able to recognize when structural differences are limited to a particular segment in the protein chain. We additionally note that lRMSD grows with chain size. However, for the purpose de novo structure prediction, lower-lRMSD conformations are more likely to be near-native than higher-lRMSD ones. When using coarse-grained representations and energy functions, a conformation with lRMSD 4 *− *5Å from the native structure is considered to have captured that structure.

### Target systems of study

Each experiment is performed on a set of 15 diverse target protein systems, shown in Table 1, ranging in size from 54 to 93 amino acids and including *α, β*, and *α/β *fold topologies. Experimentally-determined native structures are deposited for these targets in the Protein Data Bank (PDB) [61], allowing measuring the effectiveness of the proposed EA and MEA. The 15 proteins are selected to allow comparison to published results of other decoy sampling methods [27,49,62].

**Table 1:** Columns 2 *− *4 show the native PDB ID, number of amino acids and fold topology for each of the 15 target protein systems.

	Native PDB ID	Size	Fold Topology		
				**% *α***	**% *β***

1	1bq9	54	*α/β*	16	25
2	1dtdB	61	*α/β*	15	46
3	1isuA	62	*α/β*	15	19
4	1c8cA	64	*α/β*	22	48
5	1sap	66	*α/β*	30	44
6	1hz6A	67	*α/β*	31	42
7	1wapA	68	*β*	0	62
8	1fwp	69	*α/β*	30	26
9	1ail	70	*α*	84	0
10	1dtjA	76	*α/β*	26	46
11	1aoy	78	*α/β*	41	10
12	1cc5	83	*α*	47	4
13	2ci2	83	*α/β*	16	21
14	1tig	90	*α/β*	32	32
15	2ezk	93	*α*	68	0

Columns 5 and 6 break the fold topology down as the percentage of amino acids which are part of *α*-helices and *β*-sheets.

### Effectiveness of minimization in MEA over EA

This analysis compares the sampling capability of EA versus MEA when using the same energy function, AMW. In addition to a comparison in terms of a summary statistic, such as the best proximity (lowest lRMSD) to the native structure, the actual conformational ensembles are compared in terms of distributions of energy and lRMSDs to the native structure. A direct comparison is made between the two algorithms in terms of a measure of conformational diversity.

#### Comparison of lowest lRMSDs reached

**Figure 2:**
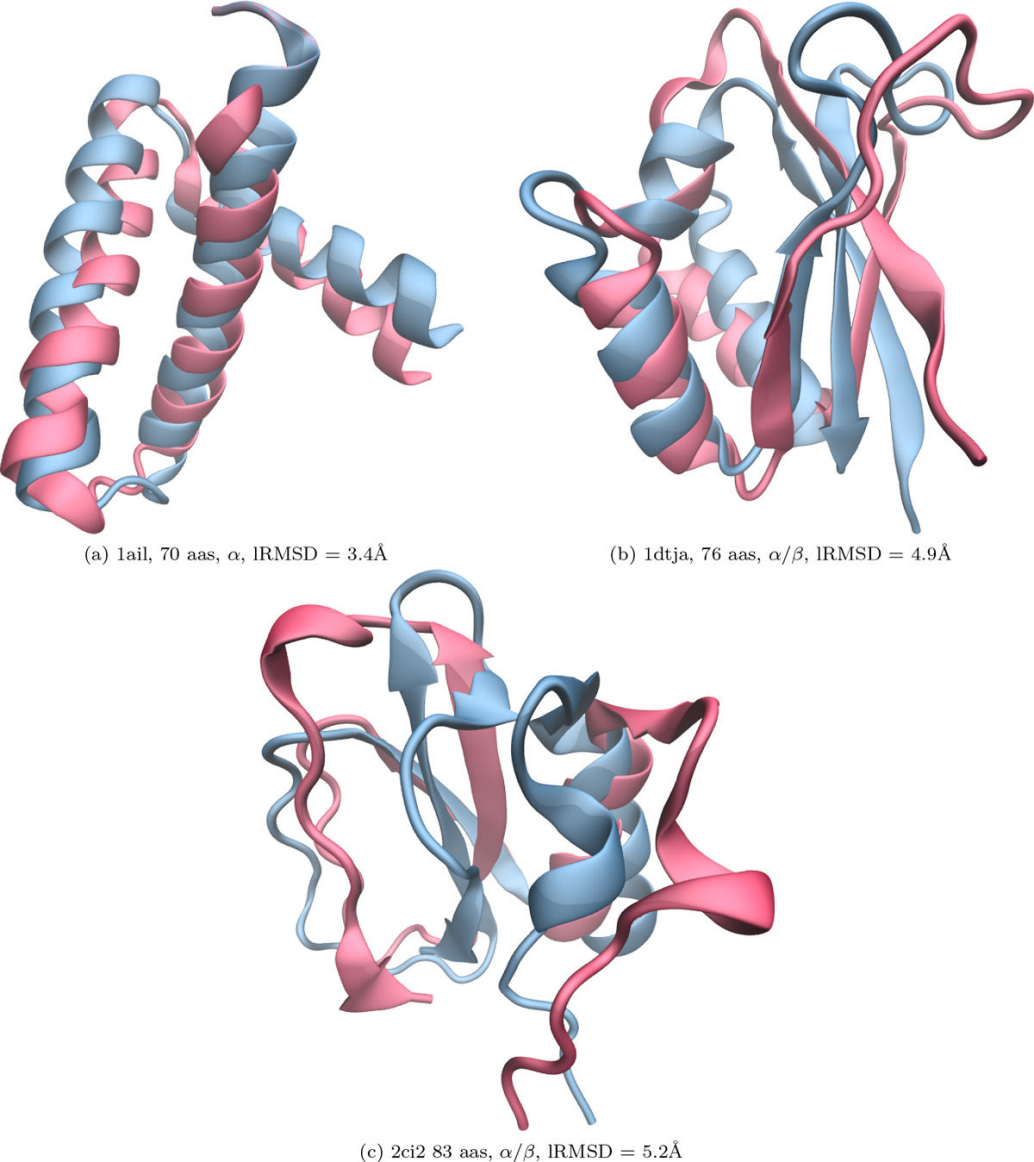
**The lowest-lRMSD conformation achieved through MEA and the AMW energy function is shown for three selected target sequences**. The chosen sequences capture the range of lowest lRMSDs achieved, varying from 3 to 5Å. The lowest-lRMSD conformation is drawn in red and is superimposed over the corresponding native structure of each sequence, which is drawn in blue. Rendering is performed with the Visual Molecular Dynamics (VMD) software [70], using the NewCartoon rendering representation which highlights secondary structures. The camera is positioned so as to highlight structural differences between the lowest-lRMSD conformation and the corresponding native structure.

Table 2 compares the performance of EA versus that of MEA in terms of proximity to the native structure. Since each algorithm is run 3 times in order to remove any artifacts due to the probabilistic nature of the algorithms, two values are reported. The lowest lRMSD to the native structure over the entire conformational ensemble Ω obtained from each run is recorded. The average and minimum values over the 3 runs are then reported in column 3 for EA and column 4 for MEA, with the minimum value shown in parentheses. We note that the ensembles in each case are obtained with the AMW energy function. Comparison of the average and minimum lowest RMSD values in these two columns shows that MEA reaches lower lRMSDs than EA. Even when focusing on the average lowest lRMSD value obtained over the independent runs, MEA achieves lower values than EA for all 15 protein systems. On 12 of these systems, the improvement over EA is by at least 1Å. This direct comparison shows that MEA is able to get closer to the native structure than EA. On a few representative protein sequences, Figure 2 shows the lowest-lRMSD conformation obtained through MEA and AMW and superimposes it over the corresponding native structure of each sequence. The viewing angle is set to highlight structural differences between the lowest-lRMSD conformation and the corresponding native structure. On the sequences where the lowest lRMSD obtained is over 4Å (Figure 2(b)-(c)), the viewing angle shows that structural differences are concentrated on the termini and are due mainly to discrepancies in configurations of loop regions. The secondary structures are generally captured, particularly on the more constrained intermediate segments of the protein chain.

**Table 2:** The lowest lRMSD to the known native structure over conformations in the output ensemble Ω*_α _*is reported for the 15 target protein systems.

	Native PDB ID	Avg(Min) Lowest lRMSD (Å)					
		**EA - AMW**	**MEA - AMW**	**MEA - Rosetta**	**FeLTr**	**ItFix**	**EdaFold**

1	1bq9	5.5(5.1)	5.2(4.8)	4.1(2.9)	6.3(5.6)	N/A	**2.0**
2	1dtdB	8.1(7.3)	7.0(6.8)	6.5(**4.7**)	7.7(7.6)	6.5	N/A
3	1isuA	7.6(7.2)	6.6(**6.4**)	7.3(7.2)	6.8(6.7)	6.5	N/A
4	1c8cA	8.6(8.5)	7.3(6.9)	7.1(6.1)	6.5(6.0)	**3.7**	N/A
5	1sap	7.9(7.2)	6.7(6.1)	5.4(5.0)	7.1(6.5)	**4.6**	N/A
6	1hz6A	6.5(5.3)	6.1(6.0)	3.4(**3.1**)	6.6(6.6)	3.8	N/A
7	1wapA	9.2(8.7)	7.7(**6.9**)	8.1(8.0)	7.8(7.3)	8.0	N/A
8	1fwp	7.6(7.2)	6.8(6.6)	6.6(**5.2**)	6.8(6.4)	8.1	N/A
9	1ail	4.9(4.1)	3.3(**3.2**)	5.5(5.1)	4.7(4.5)	5.4	N/A
10	1dtjA	8.1(7.7)	5.6(4.9)	5.4(4.5)	9.2(8.4)	N/A	**2.7**
11	1aoy	7.0(6.3)	5.4(5.1)	6.0(5.4)	5.1(**4.6**)	5.7	N/A
12	2ci2	6.9(6.5)	5.3(5.2)	4.7(3.8)	8.8(7.25)	N/A	**3.2**
13	1cc5	7.3(6.5)	5.7(**5.5**)	6.7(6.3)	6.4(6.2)	6.5	N/A
14	1tig	8.3(7.8)	6.5(6.3)	3.9(**2.6**)	10.8(10.4)	N/A	3.4
15	2ezk	6.0(4.9)	4.8(**4.4**)	5.1(4.7)	6.4(6.0)	5.5	N/A

The average and minimum lowest lRMSD obtained over 3 independent runs is shown for the Evolutionary Algorithm (EA) in column 3 and the Memetic EA (MEA) in column 4. Both of these columns are from results run with the AMW energy function. Column 5 evaluates the same MEA algorithm as column 4, but with the Rosetta energy function instead of AMW. Column 6 shows values obtained by the MMC-based FeLTr decoy sampling method [49], column 7 shows lowest lRMSDs published the Sosnick lab for the ItFix method [27], and column 8 shows lowest lRMSDs published for the EdaFold method on a subset of proteins [62].

#### Comparison of distributions of energies and lRMSDs in sampled decoy ensembles

**Figure 3:**
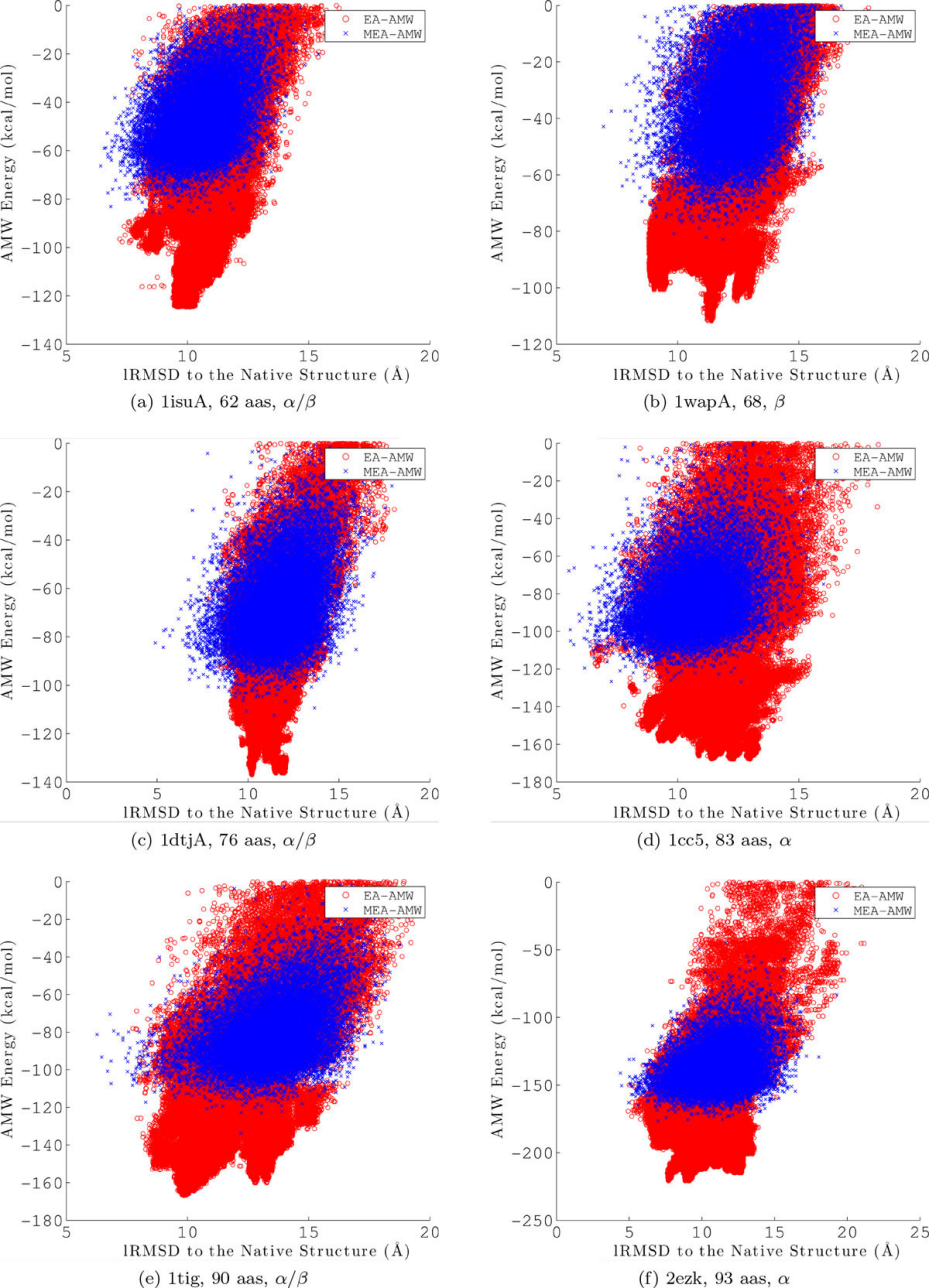
**Potential energy is plotted against lRMSD to the native structure for each conformation in the output ensemble Ω obtained by EA or MEA with the AMW energy function**. Results are combined over all 3 runs and shown for 6 selected proteins. The red "X"s are for the simple EA, and the blue circles for the MEA.

The next analysis elucidates whether better proximity to the native structure goes hand in hand with enhanced sampling of near-native conformations in MEA. Since focusing on only the lowest lRMSD sacrifices a lot of detail on the actual ensemble of conformations, we now show the entire distribution of conformations obtained by each algorithm. We do so on the combined ensemble over all 3 runs for a particular algorithm. Figure 3 plots the AMW energy of each conformation against its lRMSD to the native structure on six representative target protein systems (with native PDB ids 1isuA, 1wapA, 1dtjA, 1cc5, 1tig, and 2ezk). The systems have been chosen to span different chain lengths and native topologies. The results obtained with MEA are drawn in blue and superimposed over those obtained with EA, which are drawn in red.

Figure 3 shows that EA is able to reach much lower-energy levels than MEA. This is expected, because EA tends to converge to a few basins in the energy surface and then continues to optimize them through a highly exploitative search. In contrast, MEA tends to capture a broader view of the energy surface due to its restart mechanism described in the Methods section, which prevents it from prematurely converging to a few deep basins. This is advantageous, as many low-lRMSD conformations are associated with low energies but do not necessarily populate the lowest-energy regions in an energy surface. In fact, a growing body of research shows that many deep minima in a protein energy surface are artifacts due to the coarse-grained energy function employed and are populated by non-native conformations [39,40,49,63,64].

Figure 3 also shows that MEA yields more conformations with low lRMSDs to the native structure. On the majority of proteins, the distribution is shifted to the left, towards lower lRMSDs. A direct comparison of lRMSDs to the native structure is drawn through a histogram analysis.

**Figure 4:**
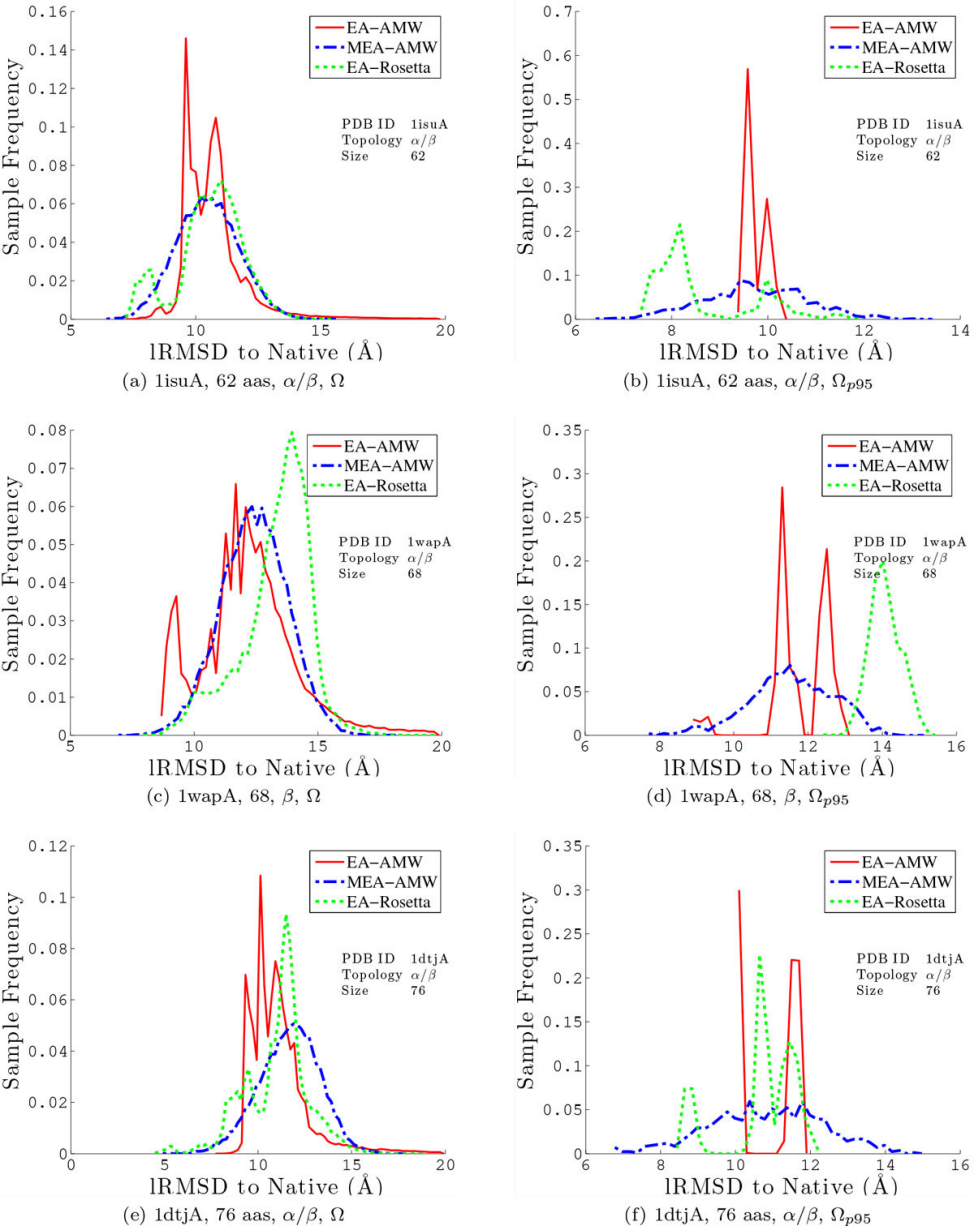
**The distribution of lRMSDs to the native structure is shown for all the conformations in the output ensemble Ω (left) and the conformations retained in the reduced ensemble Ω_*p*95_**. Results are combined over all 3 runs and shown for the first 3 of the 6 selected proteins (with corresponding PDB ids 1isuA, 1wapA, and 1dtjA). The solid red line shows the distribution obtained by the simple EA with the AMW energy function, the dashed blue line shows the distribution obtained by the MEA with the AMW energy function, and the dotted green line shows the distribution obtained by the MEA with the Rosetta energy function.

**Figure 5:**
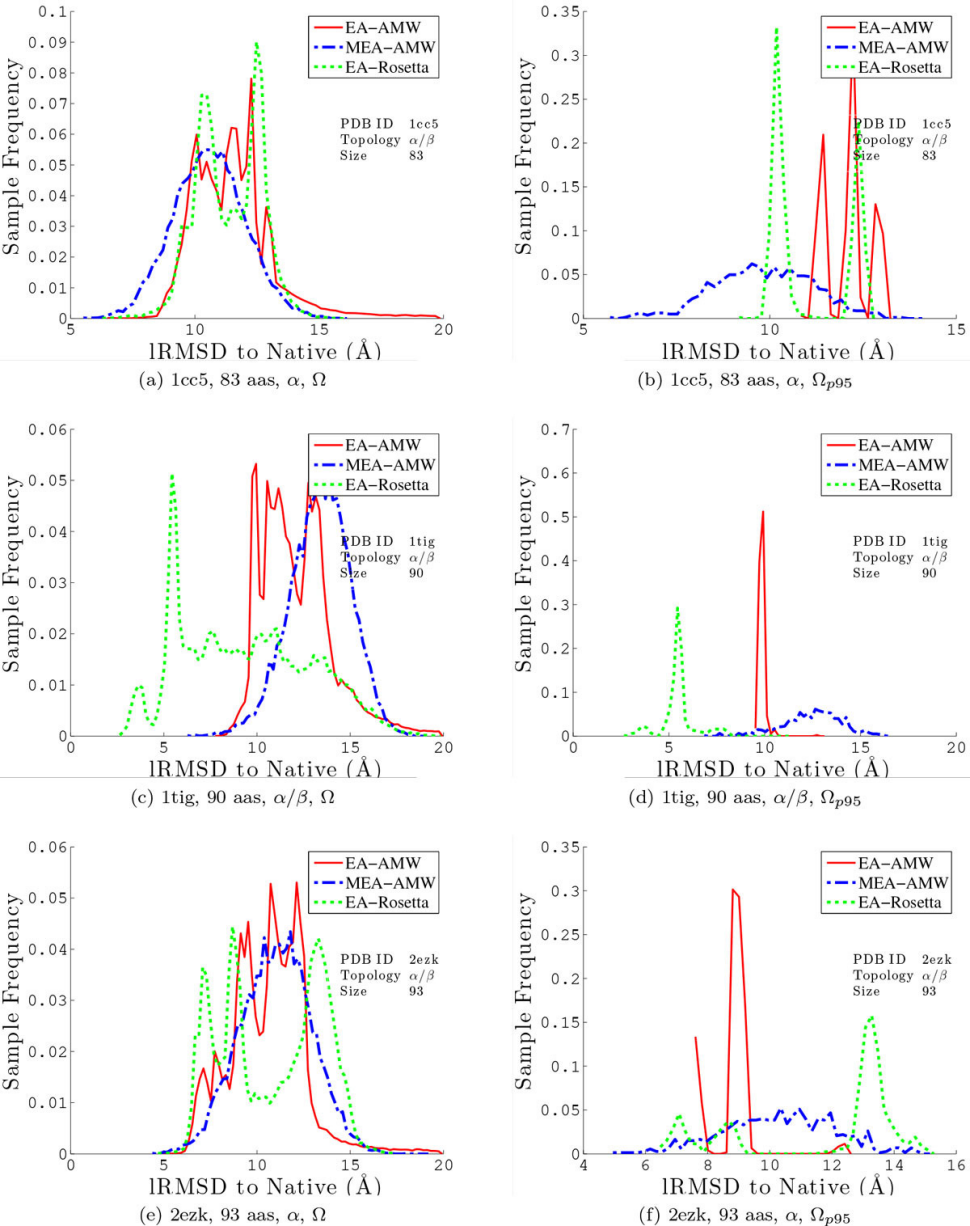
**The distribution of lRMSDs to the native structure is shown for all the conformations in the output ensemble Ω (left) and the conformations retained in the reduced ensemble Ω_*p*95_**. Results are combined over all 3 runs and shown for the last 3 of the 6 selected proteins (with corresponding PDB ids 1cc5, 1tig, 2ezk). The solid red line shows the distribution obtained by the simple EA with the AMW energy function, the dashed blue line shows the distribution obtained by the MEA with the AMW energy function, and the dotted green line shows the distribution obtained by the MEA with the Rosetta energy function.

Figures 4, 5 compare distributions of lRMSDs to the native structure obtained by MEA versus those obtained by EA on the 6 selected protein systems when using the AMW energy function. The comparison is conducted over the entire Ω ensemble and then repeated over a subset of lowest-energy conformations selected from Ω. In a complete de novo structure prediction protocol, computational constraints allow refinement of only a handful of decoy conformations in search of the true native state. While we do not conduct the second-stage refinement here, it is important to consider and compare not only the single lowest-lRMSD conformation from each method, but also the distribution of decoys that might be reasonably passed on to a second stage of refinement in a complete de novo protocol. For this purpose, here we use a simple technique that selects only conformations in the 95^th ^percentile according to energies to extract a reduced ensemble. We refer to this reduced ensemble as Ω_*p*95_.

Figures 4, 5 show the distribution of lRMSDs to the native structure over the entire Ω ensemble (shown left) and the distribution over the reduced Ω_*p*95 _ensemble (shown right). MEA and EA are directly compared in each setting. The distributions for EA are drawn using a solid red line, and those for MEA are drawn using a dashed blue line. Comparison of the distribution of lRMSDs over the entire Ω ensemble shows that low-lRMSD conformations can be found at higher-energy regions. However, focusing only on the Ω_*p*95 _ensemble consistently shows that MEA not only finds the lowest-lRMSD conformation over EA, but also samples significantly more low-lRMSD conformations than the simple EA. For 2 target proteins (1hz6A and 2ezk), the baseline EA gets lucky in one of the three runs and converges to an energy basin containing low-lRMSD conformations to the native structure. Figure 5(e)-(f) for PDB id 2ezk illustrates the rare case when the simple EA samples more low-lRMSD conformations than MEA. These results confirm that, while the simple EA can occasionally get lucky, MEA is, on average, more effective at enhancing sampling of decoy conformations near the native state. We note that a direct comparison is also drawn in Figures 4.4, 5 between MEA with AMW and MEA with Rosetta. We delay this until the comparative analysis between EA and MEA is completed in the fixed setting of one energy function, AMW.

#### Comparison of structural diversity in sampled decoy ensembles

**Figure 6:**
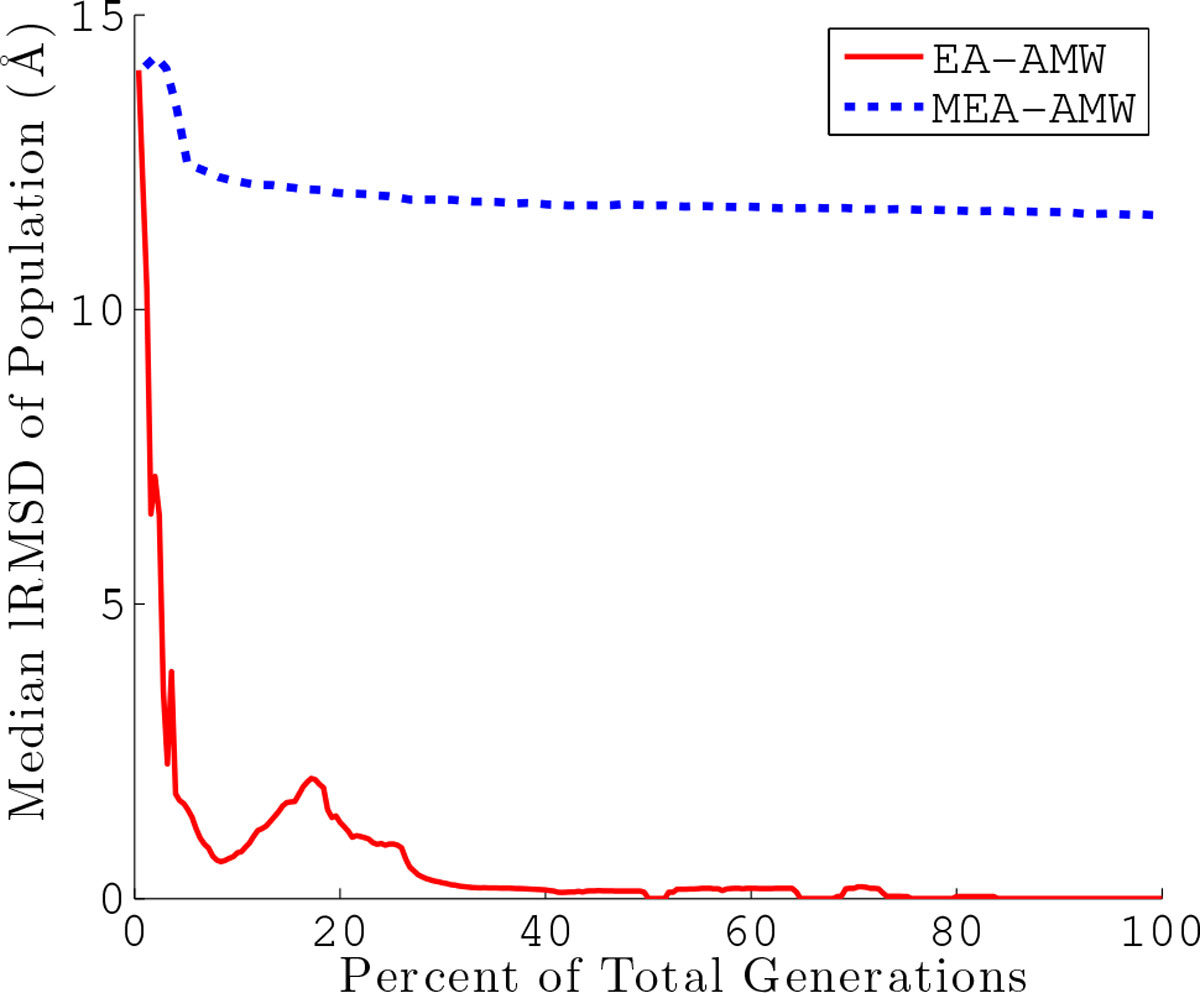
**The median lRMSD over any pair of conformations in a population is shown for each generation for a representative protein (with native PDB id **1cc5**)**. The solid red line tracks the median lRMSD per population in the simple EA using AMW, and the dotted blue line tracks this value over populations in the MEA using AMW. Since the simple EA and MEA run for a different number of generations, the generation (x-axis) is given as a percentage of the total number of generations.

The results presented above make the case that MEA is more expansive in its search due to its less greedy behavior. This results in MEA sampling more near-native conformations rather than optimizing a few non-native topologies. Here we show that the decoy ensemble obtained through MEA is also overall more structurally diverse than that obtained through EA. Measuring structural diversity is important to quantify the breadth of the exploration in these algorithms. We use a simple measure of structural diversity, the median lRMSD between any pair of conformations in a population. We track this measure over the populations in each algorithm, and show median lRMSD over populations for each algorithm in Figure 6. The solid red line shows the results for EA, and the dotted blue line shows those for MEA. We focus the comparison here to employment of the AMW energy function.

Figure 6 shows that the median pairwise lRMSD drops sharply in EA, and converges quickly to very low values. This effectively means that the population of conformations in EA loses structural diversity rather fast and never recovers. The loss of structural diversity further confirms the characterization of the behavior in EA, which greedily converges its exploration to a few energy basins. On the other hand, the median pairwise lRMSD in MEA does not experience such a sharp drop. Moreover, the structural diversity per population remains above 10Å in all subsequent populations. This suggests that MEA, due to its restart mechanism, maintains a broader view of the conformational ensemble. Coupled with the minimization component, this analysis further confirms that MEA is more appropriate at obtaining diverse decoy conformations that represent local minima in the energy surface. Effectively, MEA obtains a broad discrete representation of the energy surface relevant for the native state in terms of local minima.

### Rosetta vs. AMW energy function

Incorporating the Rosetta energy function allows drawing a direct comparison between two state-of-the-art coarse-grained energy functions in the context of a probabilistic search algorithm demonstrated by the above analysis to have high sampling capability. We first focus on comparing the lowest lRMSD values reached by MEA under each energy function and then provide more detail by comparing the ensembles in terms of distributions of lRMSDs to the native structure and distributions of lRMSDs versus energies.

Table 2 shows in column 5 the average and minimum value for the lowest lRMSD to the native structure obtained over 3 independent runs of MEA with the Rosetta energy function. Comparison of the results shown in column 4, which correspond to MEA with AMW, to those shown in column 5 for MEA with Rosetta demonstrates that the Rosetta energy function decidedly improves proximity to the native structure over AMW. Even when focusing on the average lowest lRMSDs, MEA with the Rosetta energy function obtains lower values than MEA with AMW on 9 of the 15 protein systems. On some proteins, the improvement is substantial. For instance, on proteins with native PDB ids 1bq9, 1sap, 1hz6a, 2ci2, and 1tig, the improvement is anywhere from 1 to 3Å, depending on whether one focuses on the average or minimum lowest lRMSDs obtained over 3 runs. Dramatic improvements by 3*−*4Å over average or minimum lowest lRMSDs are obtained on proteins with PDB ids 1hz6A and 1tig when using the Rosetta energy function in MEA. We note that all the proteins where the Rosetta energy function allows MEA to get closer to the native structure are of *α/β *native folds.

Closer inspection of the results in Table 2 shows that the only proteins were the AMW energy function seems to have an advantage are those that entirely or almost entirely consist of *α*-helices, like proteins with native PDB ids 1ail, 1isua, and 1cc5. This observation supports recent studies showing AMW seems well-equipped to capture the basin of all-*α *proteins but finds other topologies hard [56,65,66].

It is interesting to note that, unlike the EA and MEA with AMW, the MEA with Rosetta sometimes results in a run that reaches a lowest lRMSD substantially lower than the other runs. This trend seems to indicate that the Rosetta energy function is more attuned to the true energy landscape, possibly allowing the exploration of more areas. This also suggests that the Rosetta energy surface is more complex than AMW and can benefit from further sampling. Lastly, a large practical advantage of using the Rosetta energy function is that it is much less costly in terms of computational time, thus allowing for more exploration. This is a result of significant fine tuning of this function in the Rosetta package over the years. In the interest of a fair comparison in this paper, however, the number of energy evaluations with AMW and Rosetta were kept equivalent.

**Figure 7:**
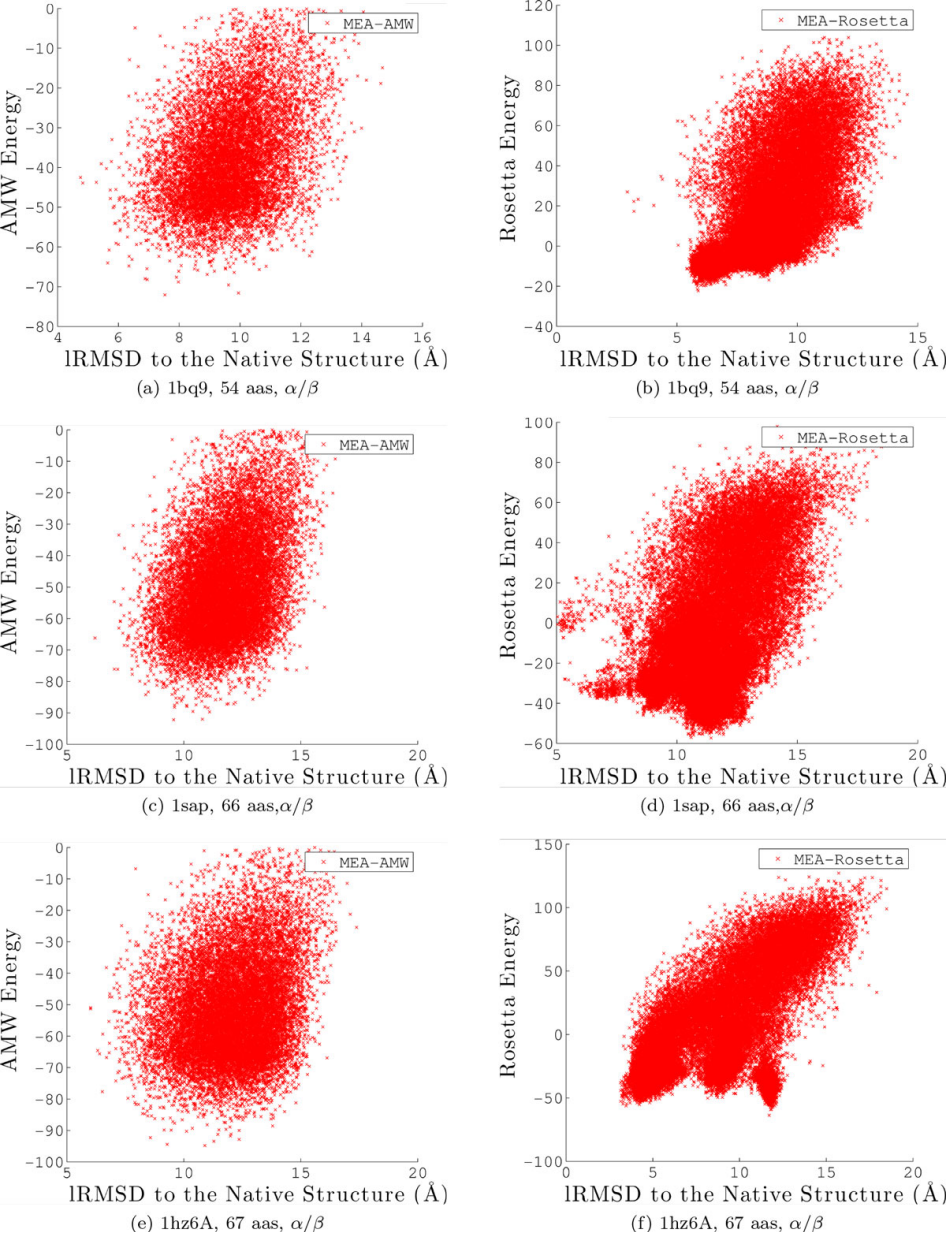
**Potential energy is plotted against lRMSD to the native structure for each conformation in the output ensemble Ω**. Results are combined over all 3 runs and shown for the first 3 of the 5 selected proteins where the Rosetta energy function seems to have an advantage over AMW (corresponding PDB ids are 1bq9, 1sap, and 1hze6A). For each selected protein, the ensemble obtained with MEA and AMW is shown to the left, and that obtained with MEA and the Rosetta energy function is shown to the right.

**Figure 8:**
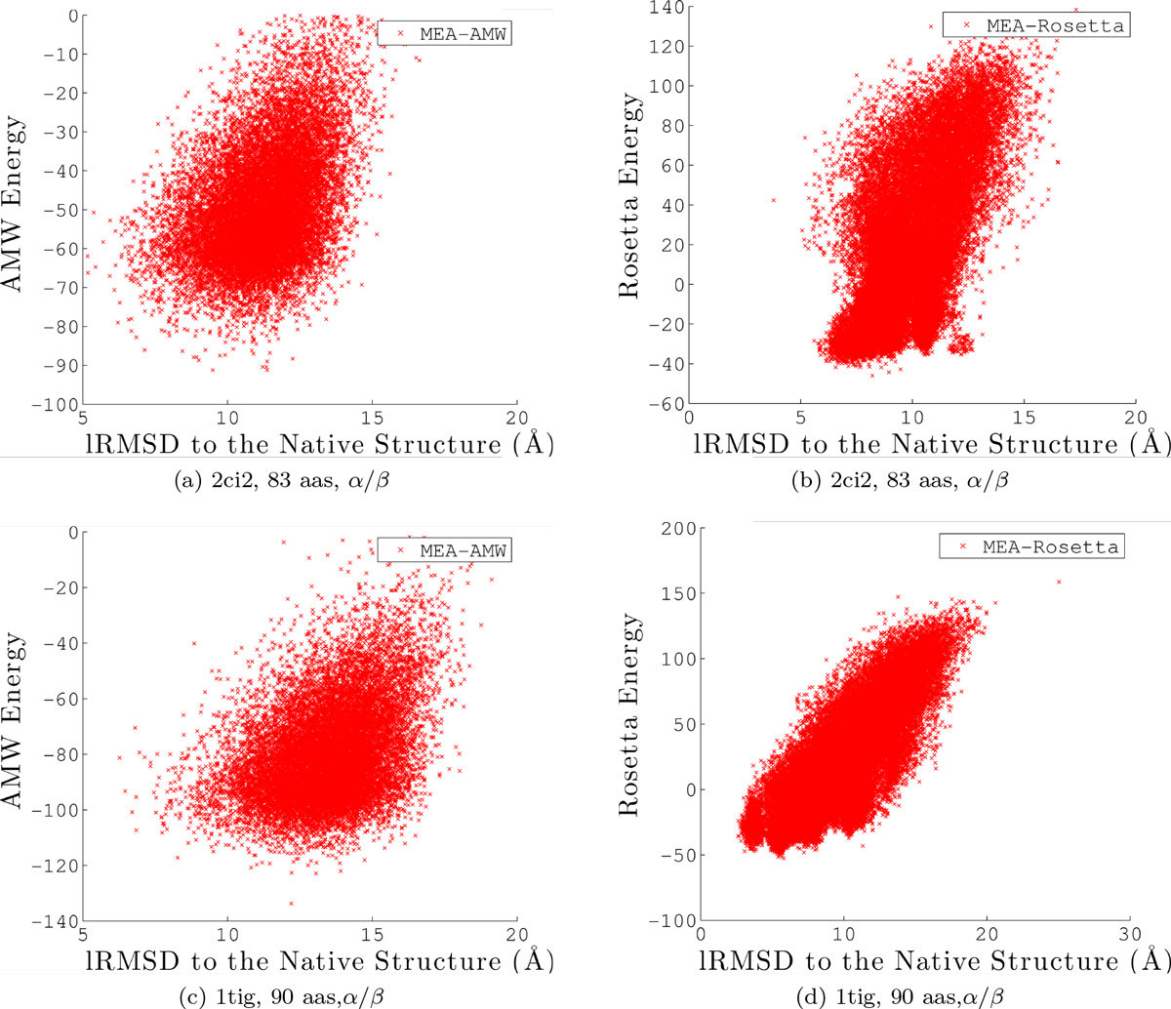
**Potential energy is plotted against lRMSD to the native structure for each conformation in the output ensemble Ω**. Results are combined over all 3 runs and shown for the last 2 of the 5 selected proteins where the Rosetta energy function seems to have an advantage over AMW (corresponding PDB ids 2ci2 and 1tig). For each selected protein, the ensemble obtained with MEA and AMW is shown to the left, and that obtained with MEA and the Rosetta energy function is shown to the right.

We analyze the *α/β *proteins on which Rosetta seems to have an advantage in more detail, in terms of the distributions of energies versus lRMSDs to the native structure for conformations obtained with MEA and AMW versus those obtained with MEA and the Rosetta energy function. Figures 7, 8 show the distribution when using AMW on the left, and the distribution when using Rosetta on the right. We note that plotting energies versus lRMSDs provides us with a view of the energy landscape explored by the algorithm under each energy function, when lRMSD to the native structure is used as the coordinate along which to distinguish sampled conformations. Strikingly, on almost all these proteins where the Rosetta energy function leads MEA closer to the native structure, the energy landscape captured with Rosetta is always more funneled. The weak funneling of the AMW energy function on these *α/β *proteins is obvious, whereas the correlation between low energies and low lRMSDs to the native structure is stronger on the Rosetta energy landscape. This is particularly the case on proteins with native PDB ids 1bq9, 1hz6A, 2ci2, and 1tig. It is worth noting, however, that the Rosetta energy landscapes do contain deep non-native minima, as can be easily seen on proteins with native PDB ids 1sap and 1hz6A.

The point on the similar inaccuracies on the AMW and Rosetta energy functions is made further through a more detailed comparative histogram-based analysis on the distribution of lRMSDs. Figures 4, 5 compare the distribution of lRMSDs obtained through MEA with the Rosetta energy function (dotted green line) to the distributions obtained with MEA and EA with the AMW energy function (red and blue lines). Taken together, comparison of lRMSDs allows making a few observations. First, employing the Rosetta energy function allows the MEA to enhance the population of low-lRMSD conformations (note, in particular, on proteins with native PDB ids 1isuA, 1dtjA, 1tig, and 2ezk). However, when focusing only on the Ω_*p*95 _ensemble, more low-lRMSD conformations are discarded when using Rosetta than the AMW energy function. These results show that the two energy functions are similarly inaccurate. While the Rosetta energy function may improve proximity to the native structure on certain folds, its energy surface, like that of AMW, contains many other deep non-native minima that would be retained if an energy-based selection scheme is used to reduce the decoy ensemble. This observation explains why many de novo protocols, including Rosetta, prefer to reduce decoy ensembles with clustering-based techniques that group decoys by structural similarity, employing the rule that an energy function may be better at preserving the width rather than the depth of the native basin.

### Comparison of proposed evolutionary search approach to state of the art

We now place the EA and MEA in context, comparing them to other decoy sampling methods in de novo structure prediction. Since we do not have access to the actual conformational ensembles produced by these methods, and lowest lRMSD to the native structure is routinely used as a measure of performance of these methods, we limit the comparison to lowest lRMSDs. All three methods chosen for comparison are MMC-based. While FeLTr [49] integrates local MMC searches into a centralized tree search structure, attempting to balance between proximity to the native structure and coverage of conformational space, the other two methods, ItFix [27] and EDAFold [62], try to gradually reduce the search space during the execution of the algorithm. All methods use molecular fragment replacement (with a slight variation in [27]), and both ItFix and EdaFold reduce the fragment configuration libraries based on distributions of fragment configurations observed among sampled conformations. In particular, EdaFold is an adaptive estimation of distribution method, updating probabilities over fragment configurations. FeLTr uses the same AMW energy function employed for our EA and MEA, and EDAFold [62] uses the Rosetta *score3 *energy function. ItFix uses the DOPE energy function [27].

Table 2 shows the lowest lRMSDs reported by FeLTr, ItFix, and EdaFold in columns 6*−*8 respectively. While FeLTr is an in-house method which we can apply to all protein systems studied here, we only have access to published results on a subset of these systems by the other two methods. N/A values in columns 7 and 8 indicate the lack of a result on a particular protein system. In addition, the reported values for EA, MEA, and FeLtr are the average and minimum over lowest lRMSDs obtained over 3 independent runs of each method, whereas the values reported for ItFix and EdaFold are only lowest lRMSDs published.

Comparing column 4 to column 6 of Table 2 shows that MEA substantially (*≥ *0.5Å) outperforms Feltr on 10 protein systems. We limit our comparison to results obtained by the MEA with AMW rather than Rosetta in the interest of a fair comparison. On 5 proteins (with native PDB ids 1bq9, 1dtjA, 1tig, 2ci2, 2ezk), MEA reaches an lRMSD to the native structure lower than 1.5Å compared to FeLTr, while FeLTr outperforms MEA only on one protein (with native PDB id 1c8cA). This suggests that the minimization step in MEA provides a distinct advantage over FeLTr, particularly in the case of longer *α*-helix and *α/β *proteins. We note this comparison was limited to MEA with AMW.

Comparing column 4 to column 7 in Table 2 shows that MEA performs comparably to ItFix. MEA finds lower or comparable lRMSDs for 6 of the 11 proteins where ItFix results are available, while ItFix finds lower lRMSDs for 2 proteins. These results are promising, as they show that employing a short greedy local search for minimization can make even a simple EA algorithm competitive for decoy sampling. Finally, comparison of columns 5 and 8 between MEA with the Rosetta energy function and EdaFold for four available proteins shows that EdaFold has a slight advantage over MEA. On two systems (with native PDB ids 1bq9 and 2ci2), the lowest lRMSD reported by EdaFold is not more than 1Å lower than the lowest lRMSD reported by MEA with Rosetta. For the system with native PDB id 1tig, MEA with Rosetta reaches a lower lRMSD than EdaFold does. Overall, by highlighting in bold the lowest lRMSD obtained by any of the methods in Table 2, one can see that the lowest lRMSD is obtained by the MEA algorithm (whether the AMW or the Rosetta energy functions are used) for 9 of the 15 protein systems. While the settings are different in terms of runtime and conformational ensembles in the methods used for comparison, this comparative analysis suggests that MEA is a powerful decoy sampling method, and further enhancements, such as estimation distribution in EdaFold, are worth pursuing.

## Conclusions

This work proposes an evolutionary search approach for decoy sampling in de novo protein structure prediction. Incorporation of state-of-the-art techniques, such as coarse graining and molecular fragment replacement, shows that even a simple EA results in decoy ensembles in good proximity to the known native structure on an extensive list of proteins. The simple EA is shown effective at optimizing a given coarse-grained energy function and reaching deep minima. However, as our analysis shows, the simple EA is highly exploitative, rapidly converging on a few basins in the protein energy surface. Once converged, the EA keeps exploiting, or drilling down in the energy surface, without making any progress towards the native structure. Conformations near the native structure are only achieved if the EA converges to a basin that happens to be near the native state.

The MEA proposed in this work remedies some of the issues of the simple EA for sampling local minima in the protein energy surface. The results show that the addition of the minimization step allows the algorithm to more effectively sample near-native conformations than a canonical simple EA. MEA is shown to be less exploitative than the simple EA. This is largely due to the additional minimization step, implemented as a greedy local search. The analysis presented above shows that the MEA quickly reaches a low-energy floor, but then uses the remaining budget to explore a breadth of conformations around that energy level while gradually reducing potential energy. This behavior is due to the fact that the greedy local search does not probe too deeply before a fragment replacement allows it to escape the current local minimum and jump to a new higher-energy conformation. Additionally, the effective moves in MEA are much larger (in terms of distance between local minima) and tend to result in similar energies. The result is that it is less likely for the entire population to converge as in the simple EA; hence, MEA is able to better maintain structural diversity in the population.

The decoy ensembles obtained through the two algorithms are compared directly in terms of structural diversity. The comparison shows that the MEA ensemble is more structurally diverse. By retaining more diversity, the MEA passes along more information to a de novo structure prediction protocol and increases the likelihood of retaining the native basin for the ensuing refinements.

Analysis of MEA also shows that the additional minimization step along with domain-specific techniques from the computational structural biology community result in evolutionary search strategies that are comparable to other state-of-the-art decoy sampling methods in the context of de novo structure prediction. High sampling capability, as attested by the structural diversity in the decoy ensemble in the MEA, is an important characteristic that makes MEA-based algorithms appealing for de novo structure prediction. Future work will investigate enhancements over the MEA proposed here to encourage greater diversity, both from an energetic and geometric point of view.

It is important to note that the consideration of two state-of-the-art energy functions illustrates the robustness and validity of the results and the conclusions in this work with respect to the comparison between MEA and EA and the comparison of these two algorithms to other ones used for decoy sampling. In particular, the elucidation of many non-native minima, particularly by MEA, shows two things. First, MEA is an algorithm with high sampling capability, a hallmark of which is exposure of non-native minima in coarse-grained energy functions [39,40,63]. Secondly, both AMW and Rosetta contain such artifacts. Comparative analysis between the results obtained under each of these energy functions shows that both are similarly inaccurate. Both functions guide MEA towards similar proximities to the known native structure. In agreement with other MMC-based studies, AMW seems to capture all-*α *native topologies better than other ones, whereas Rosetta seems to provide some improvement over AMW when *β *sheets are present.

Due to its enhanced sampling, MEA can be employed to further address current deficiencies in de novo structure prediction. For instance, the diverse decoys can be used for further development and fine tuning of coarse-grained energy functions to improve recognition and discrimination of non-native topologies. More-over, exploration capability can be increased by equipping the MEA with crossover operators, as in [67], or by replacing the energy guidance with multi-objective optimization as in [68,69].

## Competing interests

The authors declare that they have no competing interests.

## Authors' contributions

SS suggested the EA proposed in this study. BSO suggested the MEA. Both authors suggested the performance study and drafted the manuscript. AS guided the study, provided comments and suggestions on the methods and performance evaluation, and edited the manuscript.

## Declarations

The publication costs for this article were funded by the corresponding author AS through NSF Grant Nos. 1016995 and 1144106.

This article has been published as part of *BMC Structural Biology *Volume 13 Supplement 1, 2013: Selected articles from the Computational Structural Bioinformatics Workshop 2012. The full contents of the supplement are available online at http://www.biomedcentral.com/bmcstructbiol/supplements/13/S1.
